# Symbionts modify interactions between insects and natural enemies in the field

**DOI:** 10.1111/1365-2656.12586

**Published:** 2016-09-26

**Authors:** Jan Hrček, Ailsa H. C. McLean, H. Charles J. Godfray

**Affiliations:** ^1^ Department of ZoologyUniversity of OxfordSouth Parks RoadOxfordOX1 3PSUK; ^2^ Institute of EntomologyBiology Centre CASBranisovska 31Ceske Budejovice37005Czech Republic

**Keywords:** aphid, field experiment, host–parasite, host–pathogen, interactions, symbiosis

## Abstract

1. Eukaryotes commonly host communities of heritable symbiotic bacteria, many of which are not essential for their hosts' survival and reproduction. There is laboratory evidence that these facultative symbionts can provide useful adaptations, such as increased resistance to natural enemies. However, we do not know how symbionts affect host fitness when the latter are subject to attack by a natural suite of parasites and pathogens.
2. Here, we test whether two protective symbionts, *Regiella insecticola* and *Hamiltonella defensa,* increase the fitness of their host, the pea aphid (*Acyrthosiphon pisum*), under natural conditions.
3. We placed experimental populations of two pea aphid lines, each with and without symbionts, in five wet meadow sites to expose them to a natural assembly of enemy species. The aphids were then retrieved and mortality from parasitoids, fungal pathogens and other causes assessed.
4. We found that both *Regiella* and *Hamiltonella* reduce the proportion of aphids killed by the specific natural enemies against which they have been shown to protect in laboratory and cage experiments. However, this advantage was nullified (*Hamiltonella*) or reversed (*Regiella*) by an increase in mortality from other natural enemies and by the cost of carrying the symbiont. Symbionts therefore affect community structure by altering the relative success of different natural enemies.
5. Our results show that protective symbionts are not necessarily advantageous to their hosts, and may even behave more like parasites than mutualists. Nevertheless, bacterial symbionts may play an important role in determining food web structure and dynamics.

## Introduction

Most insects and many other eukaryotes carry symbiotic bacteria which are maternally inherited by their offspring. Some symbionts provide an essential service for their host, for example synthesizing nutrients which are missing in certain diets (Akman *et al*. 2002; Douglas 2014; Bennett & Moran 2015), and are termed ‘obligate’ symbionts. By contrast, ‘facultative’ symbionts are not essential for host survival and are not found in all individuals in host populations. Some facultative symbionts have no or negative effects on host fitness and spread through distorting reproduction to favour their maternal transmission (Engelstädter & Hurst 2009). Alternatively, they can be mutualistic and provide benefits to their host such as protection from natural enemies (e.g. Xie, Vilchez & Mateos 2010; Gerardo & Parker 2014; Hamilton *et al*. 2014) or against abiotic stress (e.g. Montllor, Maxmen & Purcell 2002; Heyworth & Ferrari 2015).

Facultative symbionts that benefit their hosts are particularly interesting as they provide a means through which advantageous traits can be transmitted horizontally within and between species (Jaenike *et al*. 2010; Himler *et al*. 2011; Henry *et al*. 2013). However, the benefits they provide have only been demonstrated in laboratory and cage settings (albeit with wild‐caught individuals; Jaenike *et al*. 2010) and the degree to which they help the host in the much more complex settings of the field is unclear. This is important to investigate as carrying most symbionts also imposes some costs on their hosts, for example in reduced life span (Vorburger & Gouskov 2011). Understanding how the positive and negative effects combine requires experiments in the hosts' natural ecological context. In nature, hosts are commonly subject to attack by multiple pathogens and parasites, but the vast majority of laboratory studies only consider single natural enemies (Pedersen & Babayan 2011; Johnson, de Roode & Fenton 2015). Experiments in the field are important to understand how symbionts influence the fitness of their hosts when embedded in a natural ecological community.

Pea aphids (*Acyrthosiphon pisum*) have emerged as a major model system for studying facultative symbionts (Brisson & Stern 2006). All aphids carry an obligate (primary) endosymbiont, *Buchnera aphidicola*, which provides them with essential amino acids absent from their phloem diet (Douglas 2014). In addition to *Buchnera*, seven species of facultative symbionts are found commonly in pea aphids (Henry *et al*. 2013). The pea aphid is composed of a series of host plant races which are to different extents adapted to particular host plants, and there are systematic differences in the distribution of symbionts across host races (Ferrari *et al*. 2012). The best characterized protective symbionts are *Hamiltonella defensa*, which protects aphids against parasitoids (Oliver *et al*. 2003), and *Regiella insecticola*, which is one of several endosymbionts protecting pea aphids against the aphid‐specific fungal pathogen *Pandora neoaphidis* (Scarborough, Ferrari & Godfray 2005; Łukasik *et al*. 2013) and at least one other related aphid fungal pathogen (Parker *et al*. 2013). Within a symbiont species, there is variation amongst isolates in their effects on host phenotype; for example, different isolates of *Hamiltonella* provide protection against different genera of parasitoid wasps (McLean & Godfray 2015).

Studies attempting to measure the costs of carrying facultative symbionts in aphids have produced mixed results. Under benign laboratory conditions, some studies have reported that both *Hamiltonella* and *Regiella* can be beneficial and lead to an increase in host fecundity by up to 20% (Oliver *et al*. 2008; McLean *et al*. 2011), though some costs involving reduced longevity have also been observed (Vorburger & Gouskov 2011). Laboratory aphids stressed by high temperature or humidity are more likely to die when carrying *Regiella* (Russell & Moran 2006; Parker *et al*. 2013). High temperature also imposes costs on aphids carrying *Hamiltonella* when attacked by parasitoids (Guay *et al*. 2009). Laboratory population‐cage experiments with *Hamiltonella* have shown that aphids carrying symbionts can be outcompeted by uninfected lines (Oliver *et al*. 2008). However, this disadvantage was reversed in the presence of the parasitoid against which *Hamiltonella* provides protection, which supported the idea that the protective benefits of facultative symbionts are likely to outweigh the costs (Herzog, Muller & Vorburger 2007; Oliver *et al*. 2008).

The frequency of infection by different facultative symbionts varies over space and time, and there is some evidence that this is correlated with endosymbiont function (Tsuchida *et al*. 2002; Russell *et al*. 2013; Smith *et al*. 2015). For example, the symbiont *Serratia symbiotica* protects its host against heat shock and is found at highest frequency in hot environments (Montllor, Maxmen & Purcell 2002; Henry *et al*. 2013). Aphids are commonly attacked by a wide range of natural enemies, including specialist fungal pathogens and parasitoids (van Veen *et al*. 2008; Smith *et al*. 2015). However, consistent correlations have not been found between the rate of attack of particular natural enemies and the frequency of the symbionts that confer protection against them (Smith *et al*. 2015). This is puzzling but very likely due to our lack of understanding of how symbionts affect host biology in the field (Oliver, Smith & Russell 2014).

Recently, Rothacher, Ferrer‐Suay & Vorburger (2016) placed populations of black bean aphids (*Aphis fabae*), with and without the symbiont *H. defensa*, in the field on cultivated broad bean plants (*Vicia faba*). Over the course of the season, they found a much lower parasitism rate in aphids carrying *H. defensa*, demonstrating that the protection conferred by the symbiont is indeed operational in the field. However, despite the strong protective effect, there were no differences in the population size of colonies carrying or not carrying the symbiont, suggesting that the symbiont does not necessarily provide an overall benefit to its host.

Here, we report an experimental test of whether symbionts that in the laboratory have been shown to have a protective function also benefit their hosts in the field. We worked with two facultative symbionts of the pea aphid: *R. insecticola* which confers protection against fungal pathogens and *H. defensa* which protects against parasitoids. We asked first whether the same protective function identified in the laboratory also operated in the field and second whether the aphid overall benefitted from carrying the symbiont.

## Materials and methods

### Experimental Organisms

We used two clones of the pea aphid (*A. pisum*) in our experiments. Clone R+ (laboratory code 319) was collected on *Trifolium pratense* (red clover) and naturally carried the secondary endosymbiont *R. insecticola*. Clone H+ (laboratory code 74) was collected on *Lotus pedunculatus* (greater bird's‐foot‐trefoil) and carried *H. defensa*. Both clones were of the green as opposed to red colour morph. The clones were collected near Oddington, Gloucestershire, UK, in 2003 (R+) and near Windsor, Berkshire, UK, in 2010 (H+). The clones were screened for all seven known secondary symbionts of pea aphids using diagnostic PCR (Henry *et al*. 2013). Pea aphids form host races specialized on plant species or genera, and we confirmed that our clones match their expected host race using microsatellite markers (Peccoud *et al*. 2009). Individuals from the two aphid lines were cured of their secondary symbionts using a standard antibiotic curing protocol (McLean *et al*. 2011) to establish symbiont‐free lines (termed R− and H−) more than 20 generations before the experiments began. Both the original and symbiont‐free lines were then maintained in clonal culture in the laboratory on *V. faba* (broad bean), a ‘universal’ host plant upon which most host plant races of pea aphid can feed (Ferrari *et al*. 2012).

Based on previous studies (Scarborough, Ferrari & Godfray 2005; Parker *et al*. 2013; McLean & Godfray 2015), we expected our strain of *Hamiltonella* to protect against *Aphelinus* (but not *Aphidius*) parasitoids and the *Regiella* strain to protect against fungal pathogens. We confirmed this by challenging the four aphid lines with *Aphelinus* and *Aphidius* parasitoids and the fungal pathogen *Pandora* in standard laboratory assays (Fig. S2, Supporting Information). It takes several days for the infection by fungal pathogens and parasitoids to become apparent (6 days on average for *Pandora*, 7 days for *Aphelinus* and 10 days for *Aphidius* at 20 °C). Aphids infected by fungal pathogens form a characteristic cadaver producing visible spores (Papierok & Hajek 1997) and aphids attacked by parasitoids form a distinctive dried husk, termed a ‘mummy’, when the parasitoid pupates.

### Field Experiments

**Figure 1:**
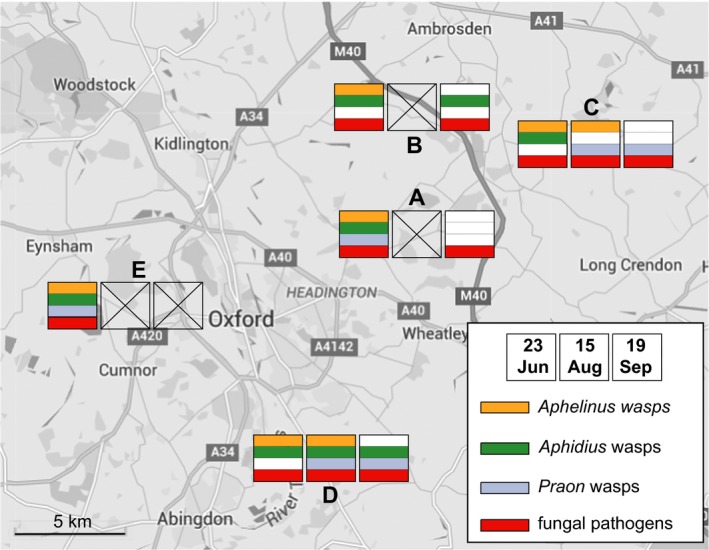
Experimental localities. Missing replicates are crossed. Coloured bars mark the presence of different natural enemies at each time and site, and a white bar means absence. For site codes, see Table S1.

Symbiont‐harbouring and symbiont‐free aphids from the two clones, feeding on their natural host plants, were placed in the field at five sites on three occasions (23rd June, 15th August and 19th September) in 2014. The five sites were all managed wet meadows spread over an area of 142 km^2^ in the south of England, UK (Fig. 1, Table S1). The aphids were left in the field for 10 days and then taken back to the laboratory to record survival and cause of mortality (parasitoid and pathogen attack).

Prior to placement in the field, the aphids were reared in 30‐cm^3^ Perspex cages on broad bean (*V. faba* cultivar The Sutton) in uncrowded conditions at 20 °C with a long day (16 h) light cycle. These conditions mimic those experienced by the summer parthenogenetic generations of aphids and allow them to complete their life cycle in 12–14 days. Experimental plants (*Trifolium pratense* and *L. pedunculatus*) were grown in pots in a glasshouse and were 6–8 weeks old when the experiments began. Twenty wingless adult aphids were placed on *Trifolium* (R+, R−) or *Lotus* (H+, H−) plants in each pot and left for 2 days to adjust to the host plant and to begin reproducing. We estimate each adult had *c*. 10 offspring in that period. Plants were then transferred to the field sites. At every site, each treatment (plant–aphid combination) consisted of a cluster of five pots with aphids, meaning that there were four clusters (20 pots) for each of the 15 site–date combinations. The four clusters were placed in a straight line *c*. 2 m parallel to a bordering hedge and *c*. 2 m apart from one another. Pots were sunk into the ground to avoid drying out and for the host plant to blend into the vegetation in the field. The order of placement of the four treatments was randomized. The experiment was blinded prior to deployment in the field so that data were collected without knowledge of the treatment.

On return to the laboratory, every plant was searched thoroughly for 4th instar and adult aphids and a total of 3244 aphids were recovered. The 10‐day exposure window in the field was chosen so that all exposed aphids would be of roughly similar size and quality. It also meant that natural enemies had the maximum time to attack the aphids, but that the majority did not kill the aphid in the field. This was necessary because parasitized aphids often wander away from the host plant prior to mummification and fungal pathogens are difficult to diagnose in the field and the chance of missing infections is high. The recovery rate of aphids from the field was 5–10%, a figure to be expected, given the aphids were exposed to ambient predation and weather. The absolute average numbers of recovered aphids for each replicate were 44·5 for aphids on *Trifolium* and 103·0 on *Lotus*; there were no significant differences between control and symbiont treatments (*P *=* *0·098 and 0·24, paired *t*‐test, in both cases, the numbers recovered from cured aphids were non‐significantly higher).

Aphids recovered from the field were put in Petri dishes containing broad bean leaves whose stalks were inserted into 2% agar to keep them fresh and kept at 20 °C under a long day (16 h) light cycle. Every 3–5 days over the following 2 weeks, the aphids were moved to fresh dishes and scored for signs of infection with fungal pathogens and parasitoid attack.

At the end of the 2‐week period, each aphid was assigned to one of four categories: survived, killed by fungal pathogen, killed by parasitoid or died from unknown reasons. Different parasitoid genera [in our study *Aphidius* and *Praon* (both Braconidae, Aphidiinae) and *Aphelinus* (Aphelinidae)] make morphologically distinctive mummies allowing their identification. We did not differentiate between fungal pathogen species, although there was at least one other species present in addition to *P. neoaphidis* based on spore morphology (Papierok & Hajek 1997). Diagnostic PCR was carried out on a subset of aphid samples to ensure that they had the expected symbiont status and that no cross‐contamination or natural immigration had occurred.

Each of the fifteen site × date combinations was treated as a replicate (hence we could not examine site by date interactions). In four cases, at least one of the treatments was destroyed by cattle, sheep or rabbit grazing, which resulted in 11 replicates in the analysis (see Fig. 1).

### Statistical Analysis

The effects of symbiont presence on aphid survival after exposure in the field were tested using a GLM logistic regression assuming a quasibinomial error distribution to account for overdispersion. We analysed the clones from the two different host plants separately. The protective effects of the symbionts were tested in the same way, but now, the response variable was the proportion of all aphid deaths that were attributable to fungal pathogens or *Aphelinus* parasitoids. This analysis is thus statistically independent of the first. For each GLM model tests, we first fitted time and site and then tested for the significance of adding a term for the presence or absence of the symbiont.

## Results

**Figure 2:**
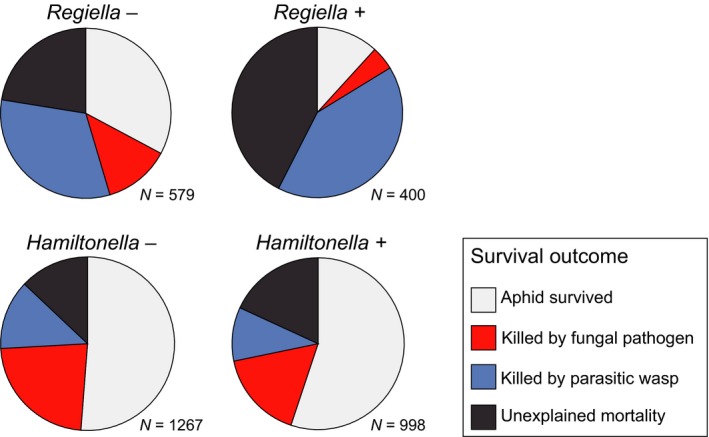
Survival outcomes for aphids exposed in the field. Following field exposure, aphids were observed for 14 days in the laboratory for signs of wasp parasitism and fungal pathogens.

**Figure 3:**
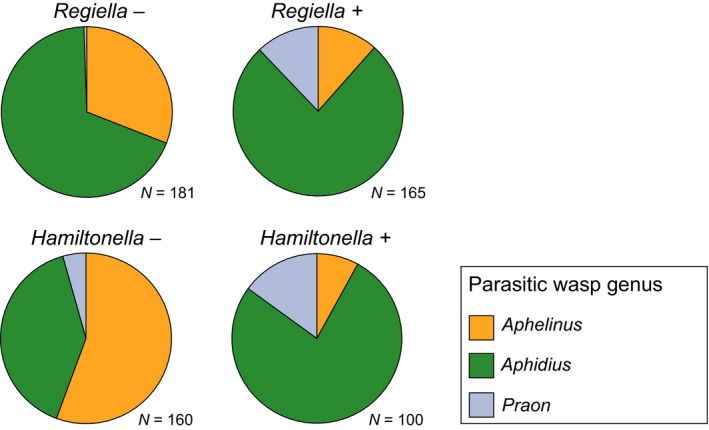
Genus composition of parasitoids developing from aphids exposed in the field.

**Figure 4:**
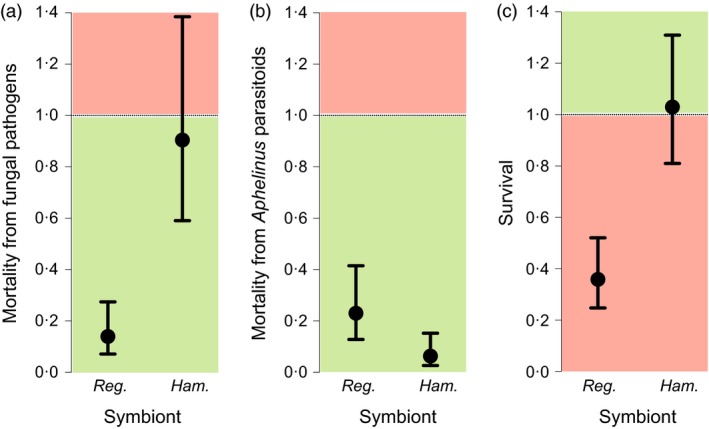
Symbiont effects. Multiplicative effect of carrying symbiont on odds ratio of (a) mortality from fungal pathogens, (b) mortality from *Aphelinus* parasitoids and (c) survival after field exposure (±SE). Values lower than one show negative influence of the symbiont on the response variable, and values greater than one show positive influence. The background colour signifies positive (green) or negative (red) influence of symbiont on host fitness for a given response variable. The odds ratios were computed using quasibinomial GLM models with main effects of time, site, and symbiont and all aphids (c) or only aphids which died (a, b) as a base.

*Regiella* protects aphids against fungal pathogens in the laboratory, and in the field, we found that symbiont presence led to a significant reduction in the proportion of aphids that succumbed to this natural enemy (*F*_1,14_ = 10·6, *P *=* *0·006; Figs 2 and 4a). To illustrate the size of the effect, the analysis predicts that in circumstances (time of year and site) where the probability of an aphid not carrying *Regiella* being killed by the fungus is 0·5 then the equivalent figure for those carrying the symbiont is 0·12 (standard error interval 0·07–0·22). The strain of *Hamiltonella* used in our experiments in the laboratory protects its host against parasitoids in the genus *Aphelinus*, and in the field, this is reflected in a lower proportion of aphids being killed by these wasps (*F*_1,14_ = 16·1, *P *=* *0·001; Figs 3 and 4b). In circumstances (time of year and site) where the probability of an aphid not carrying *Hamiltonella* being killed by *Aphelinus* is 0·5 then the equivalent figure for those carrying the symbiont is 0·06 (standard error interval 0·03–0·13). Unexpectedly, *Regiella* also reduced the proportion of aphids dying from *Aphelinus* attack, although to a lesser extent than *Hamiltonella* (*F*_1,14_ = 7·1, *P *=* *0·019; Figs 3 and 4b). This effect was not found in our laboratory assays (Fig. S2), and we suggest it is a consequence of coinfection by multiple natural enemies in the field.

Our experiments did not support the hypothesis that the presence of protective symbionts increases the overall probability of aphid survival. Having controlled for site and time, carriage of *Hamiltonella* had no effect on survival (Figs 2 and 4c; *F*_1,14_ = 0·01, *P *=* *0·905). Surprisingly, we found that carrying *Regiella* led to a significant reduction in the probability of survival (Figs 2 and 4c; *F*_1,14_ = 8·3, *P* = 0·012). The analysis predicts that in circumstances where an aphid not carrying *Regiella* would survive with probability 0·5 then the chances of surviving when carrying the symbiont is 0·26 (standard error interval 0·20–0·34).

There was considerable variation in natural enemy pressure across time and space (Fig. S1) though in all replicates some aphids died from fungal attack, and in all but one replicate parasitoid mortality occurred (Fig. 1). Parasitoid wasps are themselves attacked by hyperparasitoid wasps which in our experiments caused 15·6% parasitoid mortality. However, neither the presence of *Hamiltonella* nor *Regiella* significantly influenced hyperparasitoid hatch rate (*F*_1,14_ = 4·8, *P* = 0·072; *F*_1,14_ = 1·5, *P* = 0·26, respectively).

## Discussion

We explored whether carrying bacterial endosymbionts affected the survival of their aphid hosts after exposure to a natural suite of predators, parasitoids and pathogens in the field. The two endosymbionts we studied are generally considered to be beneficial, but their effects on pea aphid fitness have only been explored in the laboratory. We show that protective symbiosis does operate under natural conditions, as both symbionts reduced mortality from the specific enemies against which they confer protection. However, the consequences for the host were not what had been expected from laboratory and cage experiments. We found that the symbionts did not increase host fitness when faced with a complete suite of natural enemies in the field, in one case having no overall significant effect (*Hamiltonella*) and in the other reducing fitness (*Regiella*). Instead, symbiont presence affects the spectrum of natural enemies to which the host is vulnerable and hence will influence the structure and dynamics of the food web within which their host is embedded.

Our experiments show that the costs of carrying protective endosymbionts can exceed their benefits in a natural situation. Studies of the cost of endosymbiont carriage have largely been conducted under benign conditions for the aphids and typically have found no or slightly positive effects of carrying the symbiont (Castañeda, Sandrock & Vorburger 2010; McLean *et al*. 2011). Costs have tended to be observed late in life (Vorburger & Gouskov 2011) or in circumstances where the host has been stressed (Fig. S3, McLean *et al*. 2011; Parker *et al*. 2013), for example when competing for food (Oliver *et al*. 2008). However, it has generally been assumed that the benefits of protection from natural enemies would outweigh these costs in the field, and this has been supported by laboratory experiments that have shown that the competitive disadvantage of aphids carrying *Hamitonella* is reversed in the presence of parasitoids (Herzog, Muller & Vorburger 2007; Oliver *et al*. 2008). Our results from the field are therefore not what were expected from previous laboratory and microcosm experiments.

The effect of one of the symbionts we studied (*H. defensa*) on aphid population dynamics in the field has been recently tested in black bean aphids (Rothacher, Ferrer‐Suay & Vorburger 2016). Interestingly, despite very different experimental designs (e.g. different aphid and parasitoid species and different symbiont strain, study of a seasonal population trend as opposed to exposure over a single generation), both studies suggest that *Hamiltonella* does provide protection against specific parasitoids in the field but that there is no overall benefit to their hosts.

We found that there was considerable variation between sites and times of the year in the net effects of carrying each of the symbionts (Fig. S1). Even *Regiella*, which overall had a major negative effect on fitness, could in some circumstances be beneficial. Causes of aphid mortality will vary between sites and over the season (Smith *et al*. 2015), and wide variation has been observed at a single site across years (van Veen *et al*. 2008). Clearly it is possible that the environments sampled in our experiment are not representative of the average conditions experienced by symbiont‐carrying aphids in the field, something that only further field experiments can resolve. The spatio‐temporal variation in fitness may contribute to the heterogeneity in symbiont presence observed in many facultative symbiont species (Jaenike 2012). Given the extent of the experiments, we were only able to use two aphid clones, each with a single symbiont strain, and the choice of aphid clones and symbiont strains might have affected the results. Phenotypic variation between aphid clones and symbiont strains is well known, and fitness may vary according to ecological circumstances (Oliver *et al*. 2008; McLean *et al*. 2011). However, we have used natural host–symbiont pairings in which the balance of the costs and benefits should reflect a natural situation. The aphids we used for the experiments have been kept in the laboratory for several years. Because the aphids were maintained as parthenogenetic lines, we expect that their ability to adapt to novel conditions will be very limited (though note rates of mutation in symbiotic bacteria are higher than their hosts, Dunbar *et al*. 2007). The effect of any maternal effects from laboratory‐reared aphids should be mitigated by the identical treatment of symbiont and symbiont‐free lines.

Could the results be an artefact of our experimental design? The aphids in the experiment were born in the laboratory, exposed to natural enemies for 10 days in the field and then collected and brought back to the laboratory so that the cause of death could be monitored. If this procedure preferentially harmed aphids carrying symbionts, then it might have affected our results. However, evidence from laboratory studies suggests that endosymbionts in the absence of natural enemies mildly enhance survival (Oliver *et al*. 2008; McLean *et al*. 2011). Thus, we would expect any effect of time spent in the laboratory to affect survival in the opposite direction. When aphids were brought back from the field, they were transferred from their native host plants to the ‘universal’ host plant, *V. faba*. We did this in order to ensure that all host plants were of uniform quality. Again, we do not think that this is likely to have negatively affected aphids carrying endosymbionts because previous laboratory studies had shown that any cost of carrying endosymbionts is more likely to be manifest on the wild host plant (McLean *et al*. 2011). By removing aphids from the field, we also sheltered them from further natural enemies whose effects might have been influenced the outcome. However, the aphids were returned to the laboratory after the main window of attack for parasitoids and fungal pathogens, so we think it unlikely that late‐acting positive effects of endosymbionts on avoidance of these natural enemies were missed. Had the aphids been left in the field, they would have been subject to further predation but it is unlikely that predation risk is influenced by endosymbionts (Polin *et al*. 2015). Finally, any possibility of observer bias was avoided as the experiment had a blinded design.

Vertically transmitted symbionts can invade and persist in a host population if they (i) benefit the host, (ii) distort their hosts' reproduction to their own benefit or (iii) are also transmitted horizontally (Jaenike 2012). The first mechanism was thought to be most important for aphid facultative symbionts, but this is put in question by our results. Reproductive manipulation has not been found in the symbionts we studied (Moran & Dunbar 2006; Simon *et al*. 2011). Horizontal transmission does occur, but is thought to be relatively infrequent on ecological time scales (Russell *et al*. 2003). The mechanisms of horizontal transfer are unclear, although parasitic wasps can move symbionts between hosts in the laboratory (Gehrer & Vorburger 2012) and most parasitoids in the field carry and can potentially transmit, symbionts (Ahmed *et al*. 2015). Given the abundance of aphid parasitoids in most field systems, even relatively low rates of symbiont transfer could allow costly symbionts to persist. Genetic data on the distribution of symbionts across aphids provide mixed support for this hypothesis. For both *Regiella* and *Hamiltonella,* there are bacterial clades that seem to have a long association with host aphid clades but also frequent colonizations that do not give rise to long‐lasting associations (Henry *et al*. 2013). This suggests there might be relatively frequent horizontal transmission, but with occasional ‘capture’ of a symbiont within an aphid lineage.

If horizontal transmission is relatively frequent and responsible for the maintenance of symbionts in the face of host fitness costs, then these bacteria are acting more like parasites than mutualists. There is growing evidence that the division between the traditional categories of heritable symbionts – parasitic or beneficial – is more mutable than previously thought. For example, some symbiont strains of *Spiroplasma* (Xie *et al*. 2014) and *Wolbachia* (Martinez *et al*. 2015) can, in different circumstances, have positive or negative effects on host fitness. Providing protection against natural enemies can be beneficial for a symbiont that finds itself in a host attacked by a parasitoid or fungal pathogen. Modelling studies have suggested that providing protection can also influence the persistence of a symbiont or parasite in a host population (Jones, White & Boots 2007).

Hosts are commonly coinfected by multiple parasites and pathogens (Johnson, de Roode & Fenton 2015), but the outcomes of coinfections are difficult to predict. Indeed, we show that both fungal pathogens and parasitoids attack aphids at almost all sites and times of the year (Fig. 1). We show that the protection provided by symbionts affects community structure by altering the relative success of different natural enemies. Symbionts could thus be important ‘hidden players’ influencing food web structure and dynamics in a number of ways (McLean *et al*. 2016). For example, by targeting an abundant natural enemy, symbionts could provide a comparative advantage to other natural enemy species and thus prevent species domination and possibly contribute to community stability (Jones, White & Boots 2007). In addition, the presence of symbionts may affect the way food webs respond to abiotic effects (Harmon, Moran & Ives 2009). For example, symbionts are likely to be more sensitive to temperature than their hosts (Guay *et al*. 2009). Their prevalence may thus change over a temperature gradient with consequences for the structure of the food web involving the parasites and pathogens whose relative success they influence. Food web variation recorded along temperature gradients (Maunsell *et al*. 2015) might thus be affected by both direct and symbiont‐mediated effects.

We found that carriage of the symbiont protects against some natural enemies but host fitness is not improved because attack or infection by other natural enemies increases. What are these compensating natural enemies? Interestingly, they are species against which other symbionts offer protection. For example, a large proportion of *Trifolium‐*biotype aphids (both with and without *Regiella*) were killed by parasitic wasps and would therefore benefit from a symbiont protective against parasitoids (e.g. *Hamiltonella*). Carriage of multiple symbionts is not uncommon in pea aphids (clones carry on average 1·4 symbionts; Ferrari *et al*. 2012), but these insects do not carry a complete spectrum of symbionts that would afford maximum protection against all natural enemies. It is likely that the incidence of symbionts in different aphid populations reflects the history of natural enemy pressure and the costs of carrying multiple symbionts (about which we have few data from the field). It would be interesting to test the fitness of multiple symbionts, both in single and in multiple infections, in field experiments like ours.

### Conclusions

A number of symbionts have been shown to increase their hosts' resistance to specific natural enemies in laboratory and cage experiments leading to the conclusion they are mutualists. Our experimental field test shows that when hosts are exposed to a natural spectrum of parasites and pathogens, there is no overall positive effect on host fitness and in one case a strong negative effect. The symbionts do, however, reduce mortality from the specific enemies against which they confer protection. Symbionts thus modify competition between natural enemies and so may alter the structure of the food web in which their host is embedded.

## Data accessibility

Data from field exposure experiments are available from the Dryad Digital Repository: http://dx.doi.org/10.5061/dryad.3p071 (Hrček, McLean & Godfray 2016).

## Supporting information


**Table S1.** Experimental field sites.
**Fig. S1.** Differences across replicates in survival of aphids carrying and not carrying symbionts (with binomial standard errors).
**Fig. S2.** Laboratory assays of symbiont conferred protection for strains used in the field experiment.
**Fig. S3.** Endosymbionts are costly under stressful laboratory conditions.
