# Supplementary material for: Symbionts modify interactions between insects and natural enemies in the field
Source: J Anim Ecol. 2016 Sep 26;85(6):1605–12. doi: 10.1111/1365-2656.12586 (PMC5082498; doi:10.1111/1365-2656.12586)
Supplement: Supplementary file 1 — Table S1. Experimental field sites. Fig. S1. Differences across replicates in survival of aphids carrying and not carrying symbionts (with binomial standard errors). Fig. S2. Laboratory assays of symbiont conferred protection for strains used in the field experiment. Fig. S3. Endosymbionts are costly under stressful laboratory conditions. [file JANE-85-1605-s001.pdf]

Online supporting information

Table S1. Experimental field sites. BBOWT = Berkshire, Buckinghamshire and Oxfordshire Wildlife Trust.

Site code	Site	Management regime	GPS coordinates	Authority
A	Bernwood Meadows	Cut once a year in August, followed by cattle grazing	51°47'41.9"N 1°07'21.7"W	BBOWT
B	Asham Meads	Cut once a year in August, followed by sheep grazing	51°49'23.2"N 1°08'47.5"W	BBOWT
C	Rushbeds Wood meadow	Cut once a year in August	51°50'22.0"N 1°01'59.3"W	BBOWT
D	Harcourt Arboretum meadow	Cut once a year in August	51°41'01.0"N 1°12'05.4"W	Botanic Garden, University of Oxford
E	Wytham Wood meadow	Cut once a year in August	51°46'26.4"N 1°19'29.1"W	Wytham Woods, University of Oxford

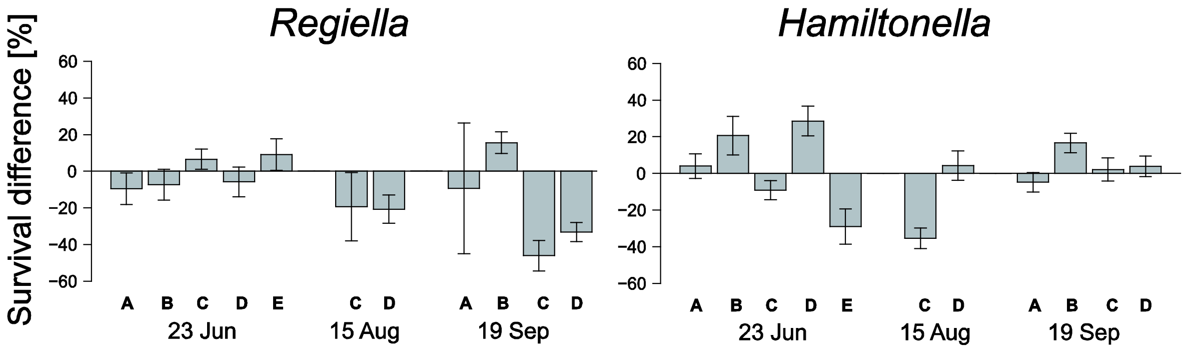

Fig. S1. Differences across replicates in survival of aphids carrying and not carrying symbionts (with binomial standard errors). Positive values indicate a benefit of symbiont carriage.

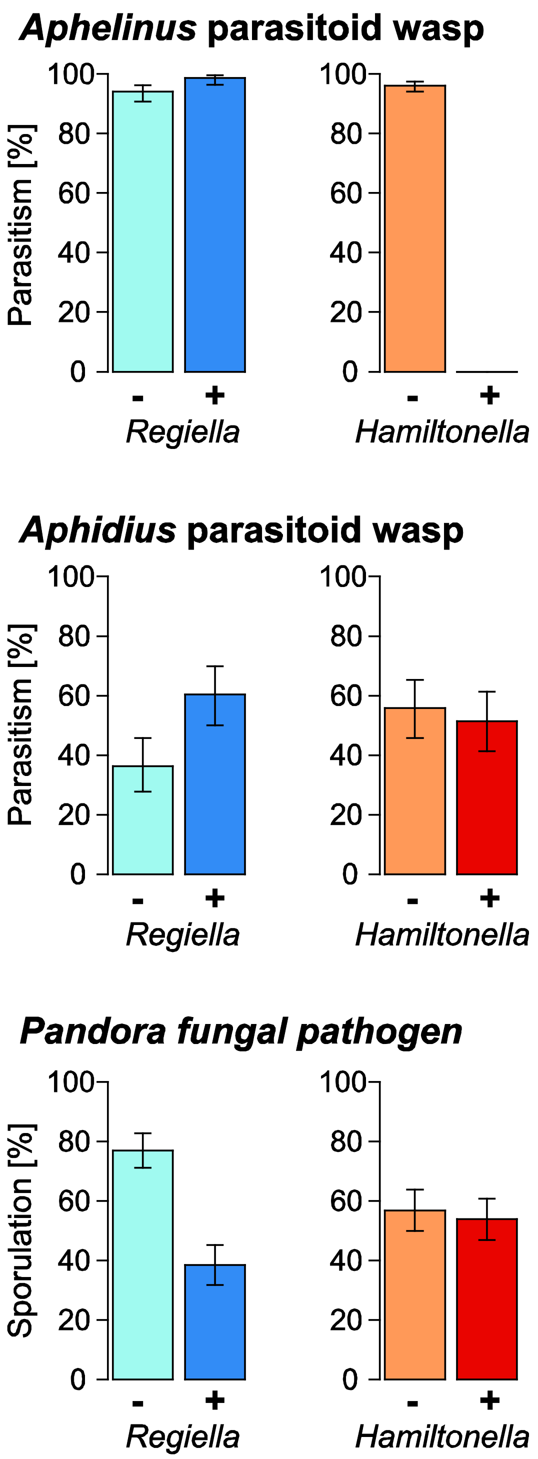

Fig. S2. Laboratory assays of symbiont conferred protection for strains used in the field experiment. Proportion of mummies or sporulating cadavers formed in the presence or absence of the symbiont. As previous studies have shown *Hamiltonella* from *Lotus* biotype aphids protects against *Aphelinus* wasps ($F_{1,8}$ = 4.9 $\times 10^{12}$, $P < 0.001$) and *Regiella* protects against the fungal pathogen *Pandora* ($\chi^2_1 = 14.2$, $P < 0.001$). The assays were conducted following published protocols with eight replicates of 15 third instar aphids exposed to parasitic wasps and followed for 11 days (McLean & Godfray 2015) and 52 adult aphids exposed to *Pandora neoaphidis* fungal spores and followed for 6 days (Parker, Garcia & Gerardo 2014).

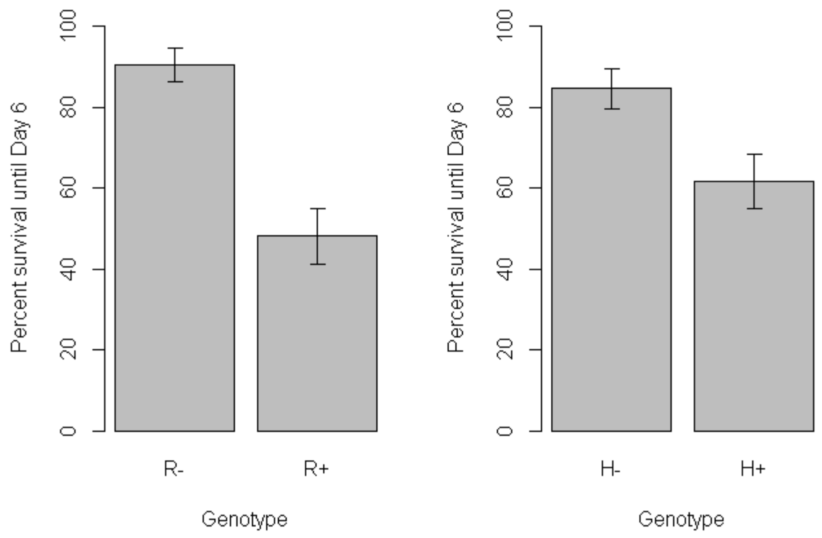

Fig. S3. Endosymbionts are costly under stressful laboratory conditions. Fifty-two adult aphids were subjected to two day high humidity (>95%) treatment at 20°C and their survival was monitored daily for six days. Both *Regiella* and *Hamiltonella* are significantly costly (Proportional Hazards Survival model, *Likelihood ratio test* = 13.6 on 1 df, $P < 0.001$ and *LRt* = 6.1, $P = 0.014$).

References

McLean, A.H.C. & Godfray, H.C.J. (2015) Evidence for specificity in symbiont-conferred protection against parasitoids. *Proceedings of the Royal Society B: Biological Sciences*, **282**, 20150977.

Parker, B.J., Garcia, J.R. & Gerardo, N.M. (2014) Genetic variation in resistance and fecundity tolerance in a natural host-pathogen interaction. *Evolution*, **68**, 2421–2429.
